# *PLOS Medicine* and Water, Sanitation, and Hygiene: A Committed Relationship

**DOI:** 10.1371/journal.pmed.1001614

**Published:** 2014-03-18

**Authors:** The PLOS Medicine Editors

## Abstract

The *PLOS Medicine* editors discuss the importance of water, sanitation, and hygiene and confirm *PLOS Medicine*'s commitment to continuing to publish WASH-related research.

*Please see later in the article for the Editors' Summary*

World Water Day falls on the 22nd of March each year. This year the focus will be on water and energy (http://www.unwater.org/worldwaterday). Throughout 2014, the United Nations and its member states will be prioritizing the important relationship between water and energy, particularly in addressing inequities for the “bottom billion” who live in slums and impoverished rural areas and who survive without access to safe drinking water, adequate sanitation, sufficient food, and energy services [1].

Water and energy have crucial direct and indirect impacts on poverty alleviation. For example, hydroelectricity is the largest renewable source for power generation, yet currently, a staggering 1.3 billion people worldwide still lack access to electricity, and roughly 2.6 billion still use solid fuels for cooking [2]. As for the statistics for access to clean water and improved sanitation, in 2011, 768 million people did not use an improved source of drinking water, and 2.5 billion people did not use improved sanitation [2].

One of the targets of Millennium Development Goal (MDG) 7, finally agreed upon in 2006, is to halve the proportion of the population without sustainable access to safe drinking water and basic sanitation between 1990 and 2015 [3]. But there are key problems with this goal. As with all of the MDG targets that include proportions of the population, the notion that a target can be deemed reached when half of the population that needs access to clean water and sanitation is still without is simply unacceptable from a human rights point of view. And although MDG 7 is frequently perceived as the “environmental MDG,” access to clean water and sanitation has an impact on all other MDGs— poverty reduction, education, gender empowerment, and reducing child and maternal mortality and infectious diseases (http://www.un.org/millenniumgoals/)—and, given the profound effects on health, should arguably be perceived as a “health” MDG.

Although the diverse role of water in energy production, as highlighted by World Water Day 2014, and in economic growth is important, the core function of clean water in improving health remains fundamental. And not just water alone, but also the three key components of the WASH agenda that have been the focus of a global campaign for over a decade—water, sanitation, and hygiene [4].

*PLOS Medicine* has long been committed to highlighting the key role of WASH in improving health. In 2009, we argued that clean water should be recognized as a human right [5]. We maintain our stance that ensuring access to clean water could substantially reduce the global burden of disease; that the privatization of water—which exploits the view that water is a commodity rather than a public good—does not result in equitable access; and that climate change, population growth, agricultural development, and industrial pollution are all leading to increasing water scarcity, threatening the quality of the current water supply. We remain of the view that a human rights framework could galvanize international recognition, concerted action, and targeted funding to help ensure that water is safe, affordable, and accessible to everyone [5].

Then in 2010 we published our landmark series (organized by Jamie Bartram, Sandy Cairncross, and colleagues) on water and sanitation (http://plos.io/1dvtfOy). The series highlighted that although water, sanitation, and hygiene are development priorities, the ambition of international policy on drinking water and sanitation was inadequate and that the active involvement of health professionals in hygiene, sanitation, and water supply was crucial to accelerating and consolidating progress for health [6], factors still pertinent to 2014. The series concluded with a rallying call for all to recognize WASH as one of the key intervention strategies for reducing morbidity, mortality, and health care costs [7]. The series also gave some targeted action points, such as how research funding agencies should consider how they could improve their support for critical research on WASH and health [7], a point that still holds true.

Our commitment to WASH has held steadfast in subsequent years. In 2011, we published an important study from Bangladesh, conducted by Stephen Luby and colleagues, which suggested that in contrast to current guidelines, handwashing with water alone could still significantly reduce childhood diarrhea, although handwashing with soap was preferable [8]. And in the same year, a study from Viet Nam, conducted by Wolf-Peter Schmidt and colleagues, showed that people living in rural villages, without access to tap water, had the highest risk of contracting dengue fever, thereby highlighting the critical role of improving water supplies in dengue control efforts [9].

The importance of sanitation was highlighted in a systematic review and meta-analysis, conducted by Kathrin Ziegelbauer and colleagues, which we published the following year [10]. This study suggested that access to sanitation was associated with a reduced risk of transmission of helminthiases to humans, leading the authors to conclude that access to improved sanitation should be prioritized alongside other interventions to achieve a sustainable reduction of the burden of helminthiases [10].

Last year, we published a key negative randomized controlled trial from India, conducted by Sophie Boisson and colleagues, that suggested that treating water with chlorine tablets had no effect in reducing diarrhea in young children and other household members, thereby questioning the health impact of household water treatment [11]. Interestingly, as with a negative randomized controlled trial from Bolivia on solar drinking water disinfection, conducted by Daniel Mäusezahl and colleagues, which we published several years ago, poor compliance with the intervention was a key issue [12]. And bringing us right up to date, we have recently published a systematic review and meta-analysis by Matthew Freeman and colleagues that highlights the importance of WASH in trachoma elimination strategies and the need to develop standardized approaches to measuring WASH in trachoma control programs [13].

Moving forward, our commitment to WASH remains central to *PLOS Medicine*, especially in light of the next chapter of international development efforts as the world transitions from the MDGs into the post-2015 Sustainable Development Goals [14], in which WASH should play a pivotal role.

The importance of water, sanitation, and hygiene has not changed over the millennia—all have, are, and always will be the foundations of human health. *PLOS Medicine* continues to welcome all appropriate WASH submissions over the coming years, particularly randomized controlled trials of WASH interventions and evaluations of implementation strategies, which help to determine how best to meet the goal of access to clean water, improved sanitation, and suitable hygiene practices for all.

## Abbreviations

MDG: Millennium Development Goal
WASH: water, sanitation, and hygiene
